# Social media, depression, and vaping susceptibility: A prospective cohort study of the combined effects in college students

**DOI:** 10.18332/tpc/230939

**Published:** 2026-10-01

**Authors:** Bara S. Bataineh, C. Nathan Marti, Alexandra Loukas, Melissa B. Harrell, Qinghua Yang, Dhiraj Murthy, Seth Schwartz, Anna V. Wilkinson

**Affiliations:** 1 Department of Epidemiology UTHealth Houston School of Public Health, Austin Austin Texas United States; 2 Department of Kinesiology and Health Education University of Texas at Austin Austin Texas United States; 3 Department of Communication Studies Bob Schieffer College of Communication, Texas Christian University Fort Worth Texas United States; 4 School of Journalism and Media, Moody College of Communication, University of Texas at Austin Austin Texas United States

**Keywords:** social media, vaping, depression, college students, Mexican American

## Abstract

**Introduction:**

Vaping has risen rapidly among Mexican-American college students in the United States (US), raising concerns about the influences of mental health and digital media. Mexican-Americans are the largest Hispanic subgroup in the US and may face unique sociocultural and environmental risk factors that increase the risk of vaping. While depression and problematic social media use (PSMU), characterized by excessive and compulsive use, are known risk factors, little research has examined their combined impact on vaping behavior. This study examined how PSMU and depressive symptoms independently and interactively influence Mexican-American college students’ susceptibility to vaping.

**Methods:**

Data were from two waves of the vaping, acculturation, and media study, a prospective longitudinal cohort study. Self-reported online survey data were collected from 828 Mexican-American college students (aged 18–29 years), across six Texas universities, who reported never vaping at baseline (Fall 2023) and completed a 1 year follow-up (Fall 2024). Mixed-effects logistic regression models tested direct effects and an interaction term for PSMU and depressive symptoms on vaping susceptibility, adjusting for sociodemographic factors, anxiety, peer vaping, exposure to pro-vaping content, and cannabis use.

**Results:**

PSMU significantly predicted greater susceptibility to vaping at follow-up at 1 year (AOR=1.45; 95% CI: 1.18–1.74). A significant negative interaction was observed (AOR=0.81; 95% CI: 0.69–0.97); simple slopes analysis showed that PSMU was associated with greater susceptibility at low (β=0.10, p<0.001) and mean (β=0.07, p<0.001) levels of depressive symptoms, but not at high levels (β=0.04; p=0.102).

**Conclusions:**

Findings indicate that the association between PSMU and vaping susceptibility varies by level of depressive symptoms among Mexican-American college students. Culturally tailored prevention strategies that integrate mental health screening with interventions targeting PSMU may help reduce curiosity and willingness to initiate vaping.

## Introduction

Electronic cigarettes (e-cigarettes) have been among the most commonly used tobacco products among young adults in the United States (US) since 2014^1,2^. Over the past 5 years, the use of e-cigarettes among college-aged young adults rose significantly from 5.4% in 2017 to 18.9% in 2022^2^. College students are at elevated risk of experimenting with e-cigarettes while simultaneously experiencing high levels of social media engagement and mental health challenges^3,4^. Despite the high prevalence, relatively little is known about the factors that influence susceptibility to vaping before initiation among college students.

E-cigarette initiation among young adults is influenced by multiple factors, including curiosity, peer pressure, appealing flavors, exposure to vaping-related content on social media, and the use of nicotine to cope with or relieve symptoms of depression and other mental health problems^3-6^. Initiation is concerning because nicotine exposure from e-cigarettes can disrupt brain development in young adults and increase the risk of addiction, substance use disorders, and long-term physical and mental health problems^6-8^. In addition, e-cigarette aerosols contain harmful chemicals that may be linked to cardiovascular and respiratory disease^9^.

Understanding why young adults become interested in vaping requires examining susceptibility to e-cigarette use, a well-established precursor to initiation. Susceptibility reflects the absence of a firm commitment not to use e-cigarettes in the future^10^. Previous research has shown that susceptibility is one of the most important predictors of e-cigarette use initiation^9,10^. For example, youth who are susceptible to e-cigarette use are approximately five times more likely to become current users within 6 months than their non-susceptible peers^11^. Even though susceptibility has been widely studied, relatively little is known about the factors that predict susceptibility among college students, limiting the development of effective vaping prevention strategies.

Among the factors believed to shape susceptibility, social media has received increasing attention. The rising popularity of e-cigarettes is strongly influenced by social media use and exposure to content that normalizes use, including advertisements, influencer promotions, and user-generated content from peers^12^. Social media platforms help normalize vaping by presenting it as socially desirable, trendy, and a safer alternative to traditional smoking^13^. Repeated exposure to social media can increase curiosity and positive attitudes toward e-cigarettes^14^.

Social media use has been associated with greater susceptibility to, experimentation with, and continued use of e-cigarettes among young people^3,12,13^. These associations may be particularly pronounced among individuals with problematic social media use^3^. Problematic social media use is conceptualized as a behavioral addiction characterized by six core components: salience, tolerance, mood modification, withdrawal, conflict, and relapse, reflecting excessive and compulsive engagement that interferes with daily functioning^14^. Unlike overall time spent or frequency of use, problematic use reflects maladaptive patterns of engagement that may reduce cognitive control and increase vulnerability to persuasive online content^15^. Consequently, individuals with problematic social media use may experience greater exposure to vaping-related content and be less able to critically evaluate messages from influencers and peers, thereby increasing their susceptibility to e-cigarette use and initiation^16^.

Mental health problems such as depression have also been associated with increased susceptibility to and initiation of e-cigarette use^4^. Depressive symptoms shape how individuals perceive, evaluate, and respond to the idea of e-cigarette use^17^. Individuals with depressive symptoms may experience impaired emotional regulation and decision-making, leading them to minimize long-term risks in favor of short-term emotional relief^18,19^.

Although problematic social media use and mental health problems, such as depression, are independently recognized as risk factors for e-cigarette use, limited research has examined whether they interact to influence susceptibility. Depressive symptoms may alter how young adults perceive and respond to vaping-related content on social media, potentially modifying the association between social media use and susceptibility to e-cigarette use^17-19^. For example, individuals experiencing higher levels of depressive symptoms may be more cognitively vulnerable to vaping persuasive messages on social media because they process information in ways that align with their mood status, making them more receptive to vaping as a coping strategy^17^. Conversely, high levels of depressive symptoms may reduce responsiveness to external stimuli, potentially attenuating the influence of vaping-related social media content on susceptibility to e-cigarette use. Individuals with elevated depressive symptoms often experience withdrawal and diminished responsiveness to environmental cues^17,18^, which may reduce the impact of vaping-related messages encountered on social media. Whether depressive symptoms strengthen or weaken the association between problematic social media use and susceptibility to e-cigarette use remains unknown.

Hispanics are the largest and fastest-growing ethnic minority population in the US, with Mexican Americans comprising nearly 59% of the Hispanic population^20,21^. Hispanic youth are particularly vulnerable to vaping due to factors such as social media exposure, limited access to mental healthcare, acculturative stress, and targeted tobacco marketing^22,23^. However, most studies aggregate Hispanic/Latiné populations, potentially masking important subgroup differences^24^. Mexican Americans may have distinct tobacco use patterns influenced by acculturation, cultural identity, and social and environmental factors, with higher lifetime smoking prevalence (36%) than individuals of Central (29%) or South American (34%) heritage^21-23^. These differences highlight the need to examine Mexican Americans as a distinct subgroup.

To our knowledge, no prospective studies have examined the independent and interactive effects of problematic social media use and depressive symptoms on susceptibility to e-cigarette use among Mexican-American college students. Thus, the current study aims to: 1) prospectively examine whether problematic social media use and depressive symptoms independently predict susceptibility to e-cigarette use 1 year later; and 2) determine whether depressive symptoms moderate the association between problematic social media use and subsequent susceptibility to e-cigarette use 1 year later among Mexican-American college students.

## Methods

### Study procedure and participants

This prospective longitudinal cohort study used self-reported data from two waves of the Vaping, Acculturation, and Media Study (VAMoS). Baseline data were collected in fall 2023, and participants were followed prospectively for 1 year, with follow-up data collected in fall 2024. Project VAMoS is an ongoing cohort study examining the impact of social media and acculturation on vaping behaviors among 1492 Mexican-American college students. Participants were recruited via email from six Texas colleges and universities: University of Texas (UT) Arlington, UT Dallas, UT El Paso, UT Rio Grande Valley, UT San Antonio, and the University of Houston System. Before participating in the study, students provided electronic informed consent and were informed that participation was voluntary and that they could withdraw at any time. Retention rate for the follow-up at 1 year was 73.3%, consistent with similar longitudinal studies of substance use among young adults^25^. Participants were compensated with a $25 e-gift card at baseline and $25 for completing the 1 year follow-up wave. The XXX Institutional Review Board (IRB) (XX) approved all study procedures, with XX IRB acknowledging and approving reliance on the XX IRB. Further details on Project VAMoS’ design and sampling methodology have been documented elsewhere^26^.

Eligibility criteria for Project VAMoS included: 1) self-identifying as Mexican American; 2) aged 18–29 years; and 3) enrolled at least part-time as a degree-seeking undergraduate student. Of the 1492 eligible students enrolled at baseline, 1093 completed the 12 month follow-up survey. Among the baseline cohort, 828 students reported never having used e-cigarettes at wave 1 and had complete data on the outcome and all variables.

### Measures

Susceptibility to e-cigarettes was adapted from measures that assess susceptibility to combustible tobacco use^10^. Participants who had never used an e-cigarette at 1 year follow-up were asked, ‘Do you think you will use e-cigarettes in the next 12 months?’ and ‘If one of your best friends were to offer you an e-cigarette, would you smoke it?’. Response options were ‘definitely not’, ‘probably not’, ‘probably yes’, or ‘definitely yes’. Participants were categorized as 0=non-susceptible if they responded ‘Definitely not’ to both items, 1=susceptible if they had any other response, and missing if they were missing on any item.

#### *Problematic social media use*/*social media addiction*

This was assessed at baseline using the 6-item Bergen Social Media Addiction Scale (BSMAS)^14^. The items measure six indicators of addiction – salience, tolerance, mood modification, relapse, withdrawal, and conflict/social impairment^14,15^. Examples include ‘I feel an urge to use social media more and more’ and ‘I spend a lot of time thinking about social media or planning how to use it’. Items were scored using a 5-point Likert scale (1=very rarely, to 5=very often). The items were summed to create a total score, ranging from 6 to 30, with higher scores indicating greater problematic social media use. The internal consistency coefficient was α=0.85.

#### Depressive symptoms

These were assessed at baseline using the PHQ-9 instrument^27^, which contains nine items that measure the frequency of depressive symptoms over the preceding 2 weeks. For example, ‘Over the last 2 weeks, how often have you been bothered by any of the following problems … little interest or pleasure in doing things, feeling down, depressed, or hopeless, etc’. The response options for each item were ‘0=not at all’, ‘1=on several days’, ‘2=on more than half of the days’, and ‘3=nearly every day’. The items are summed to create a total score, with a possible range of 0 to 27, with higher scores indicating greater severity of depressive symptoms.

#### Anxiety symptoms

These were assessed at baseline using the GAD-7 instrument^28^, which contains seven items that measure the frequency of anxiety symptoms over the preceding 2 weeks. For example, ‘Over the last 2 weeks, how often have you been bothered by … feeling nervous, anxious or on edge, not being able to stop or control worrying, etc’. The response options for each item were ‘0=not at all’, ‘1=on several days’, ‘2=on more than half of the days’, and ‘3=nearly every day’, A severity score index was calculated by summing the scores assigned to the response options for each symptom, with a possible range of 0 to 21, where higher scores indicated a greater severity of anxiety symptoms.

### Covariates

These were all assessed at baseline and were included in the analysis to adjust for potential confounding factors. The covariates included sociodemographic variables such as sex (female, male), age (years), anxiety symptoms, socioeconomic status (SES), enrollment at a university near the US–Mexico border (border institution; i.e. UT El Paso and UT Rio Grande Valley compared to other institutions), the number of close friends who use e-cigarettes (none, a few, some, most, all), and the frequency of exposure to pro-e-cigarette content on social media over the past 30 days (never, once a month, 1–2 days a week, 3–5 days a week, daily, several times a day). Socioeconomic status (SES) was assessed at baseline using the MacArthur Subjective Status Scale. (SSS). Participants selected the rung from a picture of a 10-rung ladder that represents their position in the social hierarchy relative to others in society in terms of income, education level, and occupation (1=worst off, 10=best off). High scores indicate higher placement on this social ladder^29^. Additionally, past 30 day cannabis use (0=0 days per month, 1 = ≥1 day per month) was included in all regression models.

### Statistical analysis

Descriptive statistics were calculated to summarize baseline participant characteristics. Continuous variables are presented as means and standard deviations (SDs), whereas categorical variables are presented as frequencies and percentages. Participants with missing data on the outcome or any variables included in the regression models were excluded using complete-case analysis. The proportion of missing data was low (<10%).

Mixed-effects logistic regression analyses were conducted using Stata software to examine the prospective associations between baseline depressive symptoms, problematic social media use, and susceptibility to vaping at follow-up at 1 year. To facilitate interpretation and comparability of effect sizes, continuous predictor variables were standardized (z-scores) before inclusion in the models.

First, unadjusted mixed-effects logistic regression models were fitted for each primary predictor. Next, adjusted models included age, sex, socioeconomic status (SES), border institution, anxiety symptoms, close friends who use e-cigarettes, exposure to pro-e-cigarette content on social media, and current cannabis use. To examine whether depressive symptoms moderated the association between problematic social media use and susceptibility to vaping, an interaction term (problematic social media use × depressive symptoms) was added to the adjusted model. A random intercept was included in all models to account for clustering of students within universities. Model fit was compared between models with and without the interaction term using the likelihood ratio test. To aid interpretation of a significant interaction, a spotlight (simple slopes) analysis was conducted following the approach described by Aiken and West^30^. Specifically, the association between problematic social media use and susceptibility to vaping was estimated at one standard deviation below the mean, at the mean, and one standard deviation above the mean of depressive symptoms.

Before model estimation, multicollinearity was assessed using variance inflation factors (VIF). No evidence of problematic multicollinearity was observed (all VIFs<5). Statistical significance was defined as a two-sided p<0.05.

## Results

Characteristics of participants who had never vaped at wave 1 are presented in Table 1 (n=828). Most participants were female (68.1%), with an average age of 20.9 years (SD=2.1). Approximately 37.9% of the students attended an institution near the US–Mexican border.

**Table 1:** Descriptive characteristics of the sample from the Vaping, Acculturation, and Media Study (VAMoS), a prospective cohort of Mexican-American college students enrolled at six Texas universities, Fall 2023 (N=828)

Characteristics	n (%)
**Age** (years), mean (SD)	20. 9 (2.1)
**Sex** (Female)	582 (68.1)
**SES**, mean (SD)	4.8 (1.5)
**Border institution**	314 (37.9)
**Friends vaping**	
None	398 (48.1)
A few	293 (35.4)
Some	109 (13.2)
Most	27 (3.3)
All	1 (0.1)
**Current cannabis use**	67 (8.1)
**Exposure to pro-e-cigarette social media content**	
Never	448 (54.1)
About once a month	199 (24.0)
Every few weeks	123 (14.9)
1–2 days a week	35 (4.2)
3–5 days a week	17 (2.1)
About once a day	4 (0.5)
Several times a day	2 (0.2)
**Depression**, mean (SD)	8.3 (6.7)
**Anxiety**, mean (SD)	8.5 (6.0)
**Problematic social media use**, mean (SD)	16.1 (5.8)

SES: socioeconomic status. SD: standard deviation.

Table 2 presents the unadjusted (OR) and adjusted odds ratios (AOR) from mixed-effects logistic regression models. In the main unadjusted analyses, both depressive symptoms (OR=1.33; 95% CI: 1.11–1.60, p=0.002) and problematic social media use (OR=1.50; 95% CI: 1.24–1.83, p<0.001) were associated with greater odds of susceptibility to e-cigarette use at follow-up at 1 year. After adjustment for covariates, problematic social media use remained significantly associated with greater odds of susceptibility to e-cigarette use (AOR=1.45; 95% CI: 1.18–1.77, p<0.001), whereas depressive symptoms were no longer statistically significant (AOR=1.26; 95% CI: 0.96–1.62, p=0.098). A significant negative interaction was observed between problematic social media use and depressive symptoms (AOR=0.81; 95% CI: 0.69–0.97, p=0.018) (in the case of interactions, odds ratios represent ratios of odds ratios).

**Table 2:** Mixed-effects logistic regression models predicting susceptibility to e-cigarette use, at follow-up one year from baseline, to depressive symptoms, problematic social media use, and their interaction, among Mexican-American college students in the Vaping, Acculturation, and Media Study (VAMoS), Fall 2023–Fall 2024 (N=828)

Variables	Main unadjusted effects	Main adjusted effects	Interaction adjusted effect
	OR (95% CI) p	AOR (95% CI) p	AOR (95% CI) p
**Age**		1.1 (1.0–1.19) 0.064	1.09 (0.99–1.19) 0.062
**Sex**		0.69 (0.45–1.07) 0.097	0.70 (0.45–1.09) 0.111
**Socioeconomic status (SES)**		1.15 (1.01–1.31) 0.032	1.15 (1.02–1.33) 0.032
**Border institution**		1.75 (1.16–2.64) 0.008	1.75 (1.16–2.64) 0.008
**Close friends vaping**		2.58 (2.05–3.24) 0.000	2.58 (2.05–3.25) 0.000
**Current cannabis use**		6.3 (3.56–11.19) 0.000	6.28 (3.59–11.16) 0.000
**Exposure to pro-e-cigarette content**		1.02 (0.87–1.21) 0.792	1.02 (0.87–1.21) 0.779
**Anxiety**		0.96 (0.92–1.00) 0.105	0.96 (0.92–1.01) 0.090
**Depression**	1.33 (1.11–1.60) 0.002	1.26 (0.96–1.62) 0.098	1.31 (1.00–1.74) 0.049
**Problematic social media use**	1.5 (1.24–1.83) 0.000	1.45 (1.18–1.77) 0.000	1.49 (1.22–1.83) 0.000
**Problematic social media use × Depression**			0.81^a^ (0.69–0.97) 0.018

AOR: adjusted odds ratio. CI: confidence interval. SES: socioeconomic status

a The interaction term (Problematic social media use × Depression) represents the multiplicative effect of problematic social media use and depressive symptoms on susceptibility to e-cigarette use. Statistical significance was set at p<0.05.

Simple slopes analyses for the significant interaction are presented in Table 3. At low levels of depressive symptoms (-1 SD), problematic social media use was significantly associated with susceptibility to e-cigarette use (β=0.10; 95% CI: 0.05–0.14, p<0.001). Similarly, at the mean level of depressive symptoms, problematic social media use remained significantly associated with susceptibility (β=0.07; 95% CI: 0.04–0.11, p<0.001). In contrast, at high levels of depressive symptoms (+1 SD), the association was no longer statistically significant (β=0.04; 95% CI: -0.01–0.08, p=0.102).

**Table 3:** Simple slopes analysis of the moderating effect of depressive symptoms on the association between problematic social media use at baseline and susceptibility to e-cigarette use at follow-up at one year, among Mexican-American college students in the Vaping, Acculturation, and Media Study (VAMoS), Fall 2023–Fall 2024 (N=828)

Level of depression	Marginal effects (dy/dx)^a^	SE	z	p>|z|	95% CI
−1 SD (low)	0.10	0.022	4.41	<0.001	0.05–0.14
Mean	0.07	0.018	4.03	<0.001	0.04–0.11
+1 SD (high)	0.04	0.022	1.63	0.102	−0.01–0.08

a The simple slope dy/dx represents the marginal effect, interpreted as the change in the predicted probability of susceptibility to e-cigarette use associated with a one-standard-deviation increase in problematic social media use at each specified level of depressive symptoms. Statistical significance was set at p<0.05. SD: standard deviation. SE: standard error. CI: confidence interval.

Among the covariates in the interaction model, attending a border institution (AOR=1.75; 95% CI: 1.16–2.64, p=0.008), higher socioeconomic status (AOR=1.15; 95% CI: 1.02–1.33, p=0.032), having more close friends who use e-cigarettes (AOR=2.58; 95% CI: 2.05–3.25, p<0.001), and current cannabis use (AOR=6.28; 95% CI: 3.59–11.16, p<0.001) were associated with greater odds of susceptibility to e-cigarette use.

## Discussion

The present study identified a significant association between problematic social media use and susceptibility to e-cigarette use among Mexican-American college students. This finding extends a growing body of evidence showing that greater social media use and exposure to vaping-related content increase susceptibility to, initiation of, and continued e-cigarette use among adolescents and young adults^3,16,30^. Previous studies among Mexican-American college students have similarly shown that problematic social media use is associated with more frequent daily vaping, a greater number of vaping days, and the use of higher nicotine concentrations among current e-cigarette users^3^. More recent evidence from an ecological momentary assessment (EMA) study also indicates that greater daily social media exposure is associated with increased daily e-cigarette use among Mexican-American college students^31^. These findings uniquely contribute to the literature by suggesting that problematic social media use may impact multiple stages of the vaping trajectory, from increasing susceptibility among never users to promoting more frequent use among current users.

A key contribution of this study is the significant interaction between problematic social media use and depressive symptoms. Specifically, the positive association between problematic social media use and susceptibility to e-cigarette use became weaker as depressive symptoms increased. According to Lewinsohn’s Behavioral Theory of Depression^18^, this pattern may be explained by the psychological and behavioral characteristics associated with severe depression, particularly social withdrawal, anhedonia, and reduced behavioral activation. First, social withdrawal may extend to online contexts, limiting students’ overall engagement with social media and reducing the influence of peer norms and social comparison processes. Importantly, although the models adjusted for frequency of exposure to pro-vaping social media content, the present study did not directly assess whether participants differentially attended to, processed, or were influenced by such content. Second, anhedonia and emotional blunting, hallmark features of depression, may reduce responsiveness to external rewarding or pleasurable cues more broadly^18^. Consistent with this possibility, previous research suggests that individuals with depressive symptoms may respond less strongly to emotionally salient health messages^19^. Third, reduced behavioral activation, often accompanied by high cognitive load and impairments in attention, memory, and executive functioning, may limit engagement with external social cues encountered in digital environments^18^, such as pro-vaping content and targeted advertisements, which typically heighten susceptibility to e-cigarette use. Taken together, these mechanisms remain plausible explanations but should be interpreted cautiously, given that content engagement and cue salience were not directly measured.

In contrast to individuals with severe depression, individuals with lower or moderate depressive symptoms may remain more socially engaged online, which may reflect greater receptivity to social influences and normative cues encountered on platforms such as Instagram, TikTok, and Snapchat, where vaping is often normalized^12,32,33^. This online engagement may also heighten responsiveness to observational learning and modeling processes, as outlined in Social Cognitive Theory^34^. Thus, peers and social media influencers provide behavioral models that can shape substance use intentions, making students with lower or moderate depressive symptoms more likely to adopt and reinforce these modeled behaviors^32^.

Several covariates in the model also contributed to increased susceptibility to e-cigarette use. Students attending universities near the US–Mexico border showed greater susceptibility to vaping, suggesting that border regions may represent distinct risk environments characterized by heightened exposure to tobacco marketing, cross-border access to products, and sociocultural norms that may facilitate tobacco use^35^. Also, having more friends who use e-cigarettes significantly increased susceptibility. This aligns with Social Learning Theory^34^ and a large body of studies showing that peer behaviors and norms are among the most important predictors of both e-cigarette susceptibility and initiation. Finally, current cannabis use was among the strongest predictors, which is consistent with previous research on polysubstance use patterns among adolescents and young adults^36^. Interventions should address not only individual psychological factors but also contextual factors such as border status, social influences, and other substance use behaviors that can increase susceptibility to e-cigarette use.

Broadly, the results suggest that co-occurring mental health conditions and problematic social media use do not always produce compounded risk effects. Instead, each may operate through different mechanisms or pathways that, when occurring together, can interfere with one another. Future research should investigate these underlying mechanisms to better understand the complex interactions underlying susceptibility to substance use.

### Implications

Our findings may help inform prevention campaigns and regulatory efforts aimed at college students who have problematic social media use and mild to moderate depressive symptoms by highlighting the potential role of social media marketing and peer modeling in vaping susceptibility. Campus health services may consider integrated prevention strategies that combine social media health education, routine screening for problematic social media use and depressive symptoms, and culturally responsive programming tailored to Mexican-American student populations. Although these findings do not establish causal relationships, they suggest that prevention and counseling providers may also benefit from considering students presenting with mild to moderate depressive symptoms, in addition to those with more severe depressive symptoms, when designing vaping prevention interventions. Importantly, the present findings should not be interpreted as suggesting that students with severe depressive symptoms require less attention. Rather, depressive symptoms across all levels warrant appropriate mental health screening, referral, and treatment.

### Limitations

The study has limitations. First, participants were recruited from six universities in Texas. Restricting recruitment to selected universities may have introduced selection bias and limited the generalizability of the findings to students attending other institutions or geographical regions. Additionally, the use of a gift card incentive may have influenced participation and also led to potential selection bias. Second, the study relied on self-report measures, which are subject to recall bias and social desirability bias. Additionally, symptoms of depression were assessed using the PHQ-9, which is a validated screening tool and predictor of depression but is not diagnostic^27^. Lastly, although adjustments were made for known confounders**,** residual confounding due to unmeasured variables may have influenced the observed associations. Potential unmeasured confounders include personality traits (e.g. sensation seeking), family history of substance use or mental health conditions, and environmental factors that were not assessed.

## Conclusions

This study highlights that problematic social media use is associated with greater susceptibility to vaping among Mexican-American college students and that this association varies by the level of depressive symptoms. Although the findings are limited by the use of a sample from only Texas colleges and self-reported measures, they provide important evidence for understanding vaping risk in this population. Future studies should replicate these findings in more diverse populations, incorporate clinical assessments of depression, and examine the mechanisms through which problematic social media use and mental health jointly influence vaping susceptibility.

## References

[1] National Center for Chronic Disease Prevention and Health Promotion (US) Office on Smoking and Health (2016). E-Cigarette Use among Youth and Young Adults: a Report of the Surgeon General.

[2] Patrick ME, Schulenberg JE, Miech RA, Johnston LD, O’Malley PM (2023). Monitoring the future panel study annual report: national data on substance use among adults ages 19 to 60, 1976-2022.

[3] Bataineh BS, Hébert ET, Loukas A (2024). Problematic social media use and vaping among Mexican-American college students. Digit Health.

[4] Bataineh BS, Wilkinson AV, Sumbe A (2023). Depressive symptoms and the age of initiation of tobacco and marijuana use among adolescents and young adults. Drug Alcohol Depend.

[5] Luzius A, Dobbs PD, Jozkowski KN (2020). College students’ reasons for using different e-cigarette products: a mixed methods analysis. J Am Coll Health.

[6] Wilkinson AV, Chen B, Swann AC (2024). Use of cigarettes and e-cigarettes, impulsivity, and anxiety: influences on suicidal ideation among youth and young adults in Texas. Nicotine Tob Res.

[7] Bataineh BS, Wilkinson AV, Case KR (2021). Emotional symptoms and sensation seeking: implications for tobacco interventions for youth and young adults. Tob Prev Cessat.

[8] Leslie FM (2020). Unique, long-term effects of nicotine on adolescent brain. Pharmacol Biochem Behav.

[9] Banks E, Yazidjoglou A, Joshy G (2023). Electronic cigarettes and health outcomes: epidemiological and public health challenges. Int J Epidemiol.

[10] Pierce JP, Choi WS, Gilpin EA, Farkas AJ, Merritt RK (1996). Validation of susceptibility as a predictor of which adolescents take up smoking in the United States. Health Psychol.

[11] Bold KW, Kong G, Cavallo DA, Camenga DR, Krishnan-Sarin S (2017). E-cigarette susceptibility as a predictor of youth initiation of e-cigarettes. Nicotine Tob Res.

[12] Jung S, Murthy D, Bateineh BS, Loukas A, Wilkinson AV (2024). The normalization of vaping on TikTok using computer vision, natural language processing, and qualitative thematic analysis: mixed methods study. J Med Internet Res.

[13] Vogel EA, Ramo DE, Rubinstein ML (2021). Effects of social media on adolescents’ willingness and intention to use e-cigarettes: an experimental investigation. Nicotine Tob Res.

[14] Griffiths MD (2005). A “components” model of addiction within a biopsychosocial framework. J Subst Use.

[15] Dailey SL, Howard K, Roming S, Grimes T (2020). A biopsychosocial approach to understanding social media addiction. Hum Behav Emerg Technol.

[16] Zheng X, Li W, Wong SW, Lin HC (2021). Social media and e-cigarette use among US youth: longitudinal evidence on the role of online advertisement exposure and risk perception. Addict Behav.

[17] Tercyak KP, Goldman P, Smith A, Audrain J (2002). Interacting effects of depression and tobacco advertising receptivity on adolescent smoking. J Pediatr Psychol.

[18] Lewinsohn PM, Friedman RJ, Katz MM (1974). The psychology of depression: contemporary theory and research.

[19] Stevens EM, Lee DN, Stevens H, Sadasivam RS (2024). The role of mood in shaping reactions to smoking cessation messages among adults who smoke: a multimodal investigation. BMC Public Health.

[20] New estimates highlight differences in growth between the U.S (June 27, 2024). Hispanic and Non-Hispanic populations. US Census Bureau. https://www.census.gov/newsroom/press-releases/2024/population-estimates-characteristics.html.

[21] (2026). Pew Research Center. https://www.pewresearch.org/.

[22] Sanders EC, Evans S, Budenz A, Frias NY, Byrnes S, Macario E (2024). Identity, acculturation, and e-cigarette use among bicultural hispanic youth and young adults. Health Equity.

[23] Silmi KP, Castillo V, Segura N (2025). Social determinants of alcohol and tobacco use among hispanic adolescents: a scoping review. Front Psychiatry.

[24] Parrinello CM, Isasi CR, Xue X (2015). Risk of cigarette smoking initiation during adolescence among US-born and non–US-born Hispanics/Latinos: the Hispanic community health study/study of Latinos. Am J Public Health.

[25] Yeterian JD, Dow SJ, Kelly JF (2012). Ensuring retention in longitudinal studies: a practical evaluation of an intensive follow-up protocol and suggested adaptations. Int J Soc Res Methodol.

[26] Bataineh BS, Marti CN, Murthy D (2025). Vaping, acculturation, and social media use among Mexican American college students: protocol for a mixed methods web-based cohort study. JMIR Res Protoc.

[27] Kroenke K, Spitzer RL, Williams JB (2001). The PHQ-9: validity of a brief depression severity measure. J Gen Intern Med.

[28] Spitzer RL, Kroenke K, Williams JB, Löwe B (2006). A brief measure for assessing generalized anxiety disorder: the GAD-7. Arch Intern Med.

[29] Goodman E, Adler NE, Kawachi I, Frazier AL, Huang B, Colditz GA (2001). Adolescents’ perceptions of social status: development and evaluation of a new indicator. Pediatrics.

[30] Aiken LS, West SG (1991). Multiple regression: testing and interpreting interactions.

[31] Hébert ET, Bataineh BS, Marti CN, Yang Q, Loukas A, Wilkinson AV (2026). Daily associations between social media exposure and e-cigarette use among Mexican American college students. Subst Use Misuse.

[32] Pokhrel P, Ing C, Kawamoto CT, Laestadius L, Buente W, Herzog TA (2021). Social media’s influence on e-cigarette use onset and escalation among young adults: what beliefs mediate the effects?. Addict Behav.

[33] Bataineh BS, Talavera-Garza L, Loukas A (2026). Factors influencing vaping behaviors based on social cognitive theory: a qualitative study among Mexican-American college students. Subst Use Misuse.

[34] Bandura A (1974). Social foundations of thought and action: a social cognitive theory.

[35] Herrera AL, Wilkinson AV, Cohn EA, Perry CL, Fisher-Hoch SP (2018). Exposure to e-cigarette marketing and product use among Mexican American young adults on the US-Mexico border: a pilot study. Cogent Med.

[36] Sun R, Mendez D, Warner KE (2023). The association between cannabis use and subsequent nicotine electronic cigarette use among US adolescents. J Adolesc Health.

